# Body Adiposity Index Utilization in a Spanish Mediterranean Population: Comparison with the Body Mass Index

**DOI:** 10.1371/journal.pone.0035281

**Published:** 2012-04-09

**Authors:** Angel A. López, Mey L. Cespedes, Teofila Vicente, Matias Tomas, Miguel Bennasar-Veny, Pedro Tauler, Antoni Aguilo

**Affiliations:** 1 Prevention of Occupational Risks in Health Services, Balearic Islands Health Service, Palma, Spain; 2 Prevention of Occupational Risks, Correos, Valencia, Spain; 3 Prevention of Occupational Risks, Balearic Islands Government, Palma, Spain; 4 Research Group on Evidence, Lifestyles & Health, Balearic Islands University, Palma, Spain; University of Colorado Denver, United States of America

## Abstract

**Background:**

Body fat content and fat distribution or adiposity are indicators of health risk. Several techniques have been developed and used for assessing and/or determining body fat or adiposity. Recently, the Body Adiposity Index (BAI), which is based on the measurements of hip circumference and height, has been suggested as a new index of adiposity. The aim of the study was to compare BAI and BMI measurements in a Caucasian population from a European Mediterranean area and to assess the usefulness of the BAI in men and women separately.

**Research Methodology/Principal Findings:**

A descriptive cross-sectional study was conducted in a Caucasian population. All participants in the study (1,726 women and 1,474 men, mean age 39.2 years, SD 10.8) were from Mallorca (Spain). Anthropometric data, including percentage of body fat mass obtained by Bioelectrical Impedance Analysis, were determined. Body Mass Index (BMI) and BAI were calculated. BAI and BMI showed a good correlation (*r* = 0.64, *p*<0.001). A strong correlation was also found between BAI and the % fat determined using BIA (*r* = 0.74, *p*<0.001), which is even stronger than the one between BMI and % fat (*r* = 0.54, *p*<0.001). However, the ROC curve analysis showed a higher accuracy for BMI than for the BAI regarding the discriminatory capacity.

**Conclusion:**

The BAI could be a good tool to measure adiposity due, at least in part, to the advantages over other more complex mechanical or electrical systems. Probably, the most important advantage of BAI over BMI is that weight is not needed. However, in general it seems that the BAI does not overcome the limitations of BMI.

## Introduction

Obesity is a chronic, multifactorial and complex disease which is defined as an excess in body fat. Due to continuous increase in prevalence, obesity has become one of most important public health problems in the world. The increase in prevalence of obesity involves an increase in the prevalence of several obesity-related comorbidities and an increase in mortality rates [1]-[9]. Thus, body fat content and, especially, the fat distribution or adiposity are used as indicators of health risk. Consequently, diagnosis and treatment of obesity is a major health issue, which many times overwhelms the medical systems and increases the economic costs [10]–[16].

Several techniques have been developed and used for assessing and/or determining body fat or adiposity. These methodologies include, among others, body mass index (BMI), waist circumference, waist-hip ratio, skinfold thickness, dual-energy X-ray absorption (DXA) and hydrostatic densitometry. However, some of these techniques are too complex and expensive to be applied on a routine or regular basis. Furthermore, some of these methodologies are clearly inaccurate because of their intra and inter-observer variability [17]. The introduction of bioelectrical impedance could suppose a significant improvement in the methodology developed for assessing body fat. In fact, bioelectrical impedance has been considered a valid alternative for measuring body fat because it does not present some of the limitations indicated previously for the other techniques. In addition, bioelectrical impedance has been validated against reference methods [18], [19].

Increased body fat is supposed to be accompanied by increased total body mass, in both men and women. Thus, indices of relative weight are commonly used to diagnose obesity [3]-[6], [20], [21]. BMI is the most widely used and accepted index to characterize obesity in individuals [22], [23]. However, BMI presents some important limitations which could lead to, for example, classify individuals with high muscle mass as overweight or obese and, on the other hand, subjects with a high percentage of fat can present a BMI within the normal range [24]–[26]. Furthermore, BMI can not be determined in places where it is difficult to get an accurate measure of weight, as in developing countries.

Bergman et al. suggested a new index, the body adiposity index (BAI) based on the measurements of hip circumference and height. Thus, it can be measured in places where the accurate measurement of weight is difficult. This index showed a high correlation with body fat measured using DXA. In their study, Bergman et al. also found that this correlation was higher than the one between BMI and body fat measured using DXA when men and women were considered together. However, this study was conducted only in two U.S. ethnic populations, African Americans and Mexican Americans, but not in Caucasians [7].

As indicated previously, BAI calculation involves the use of hip circumference. It has been suggested that hip circumference captures male–female differences in adiposity better than the BMI [7]. Thus, in this sense, the utilization of hip circumference could suppose an important conceptual advantage of the BAI over BMI. Taking into account this observation, it is expected that the BAI would be better in predicting body fat in men and women separately.

The aim of the study was to compare BAI and BMI measurements in a Caucasian population from an European Mediterranean area and to assess the usefulness of the BAI in men and women separately. Furthermore, we aimed to correlate BAI with measures obtained using bioelectrical impedance and to demonstrate the usefulness of the BAI in the routine clinical practice.

## Materials and Methods

### Subjects and Study Protocol

A descriptive cross-sectional study was conducted in a Caucasian population. All subjects were from Mallorca (Spain) and belong to different productive sectors (public administration, health department, post offices). Subjects participating in the study were systematically selected during their work health periodic examinations. Every day the first and the last examined worker were invited to participate in the study. 3,223 workers were invited to participate in the study. However, 23 refused to participate, being the final number of participants 3,200 (99.3%), with 1,726 women and 1,474 men. The mean age of participants in the study was 39.2 years (SD 0.19). Participants were informed of the purpose of this study before they provided written consent to participate. Following the current legislation, members of the Health and Safety Committees were informed as well. The study protocol was in accordance with the Declaration of Helsinki and was approved by the Institutional Review Board (GESMA). After acceptance, a complete medical history, including family and personal history, was recorded. Occupational data was also recorded. This study was conducted between January 2008 and December 2010. The following inclusion criteria were considered: age between 18 and 65 years (working age population), agreement to participate in the study and to be gainfully employed. Subjects who did not meet any of the inclusion criteria and those who refused to participate were excluded from the study.

### Measurements and Calculations

#### Anthropometrics

All anthropometric measurements were made in the morning, after an overnight fast, at the same time (9 a.m.), and according to the recommendations of the International Standards for Anthropometric Assessment (ISAK) [27]. Furthermore, all measurements were performed by well trained technicians or researchers to minimize coefficients of variation. Each measurement was made three times and the average value was calculated. Weight and height were determined according to recommended techniques mentioned above. Body weight was measured to the nearest 0.1 kg using an electronic scale (Seca 700 scale, Seca gmbh, Hamburg). Height was measured to the nearest 0.5 cm using a stadiometer (Seca 220 (CM) Telescopic Height Rod for Column Scales, Seca gmbh, Hamburg).). BMI was calculated as weight (kg) divided by height (m) squared (kg/m^2^). Criteria used to define overweight were the ones of the World Health Organization (WHO) [28], which considers obesity when BMI ≥ 30 kg/m^2^. Abdominal waist and hip circumferences were measured using a flexible steel tape (Lufkin Executive Thinline W 606). The plane of the tape was perpendicular to the long axis of the body and parallel to the floor. Waist circumference was measured at the level of the umbilicus and the superior iliac crest. The measurement was made at the end of a normal expiration while the subject stood upright, with feet together and arms hanging freely at the sides. Hip circumference was measured over nonrestrictive underwear or light-weight shorts at the level of the maximum extension of the buttocks posteriorly in a horizontal plane, without compressing the skin.

The body adiposity index (BAI) was calculated using the equation suggested by Bergman and colleagues, BAI  =  ((hip circumference)/((height)^1.5^)-18).

Percentage of body fat mass was obtained by Tetrapolar Bioelectrical Impedance Analysis (BIA) system (BF-350, Tanita Corp, Tokyo, Japan). BIA measurements were carried out at 50 kHz with a 0.8 mA since wave constant current under standard conditions Whole-body composition was estimated using equations provided by the BIA manufacturer for all participants [27]. The reliability and validity of this system has been proved in Caucasian populations. BIA measurement using this methodology has been described in detail previously [29]. Subjects stood on the metal contacts in bare feet, and body fat mass was determined. This measurement was repeated twice, and the average value was obtained.

### Statistical Analyses

All the data were tested for their normal distribution (Kolmogorov–Smirnov test). Results are expressed as means and standard deviations (SD) and, when required, in percentages. Student t test for unpaired data was used to evaluate differences in anthropometric characteristics between genders (Table 1). The existence of significant bivariate correlations among parameters such as BAI, BMI, height, weight, hip circumference and % fat determined by BIA was ascertained by means of determining Pearson correlation coefficients. The statistical method of ROC curves (Receiver operating characteristic curves), which allows the evaluation of several cutoff points for different pairs of sensitivity and specificity, was used to determine the BAI breakpoint. Cutoff values were derived mathematically from the ROC curves, using the point on the ROC curve with the lowest value for the formula: (1-sensitivity)^2^ + (1-specificity)^2^. The positive predictive value (PPV) and the negative predictive value (NPV) were also determined. A p value < 0.05 was considered statistically significant. Statistical analysis was carried out using IBM SPSS Statistics 19.0 software (SPSS/IBM, Chicago, IL, USA).

**Table 1:** Anthropometric characteristics of participants in the study.

	All(n = 3,200)	Men (n = 1,474)	Women (n = 1,726)	*p* value
	Mean (SD)	Mean (SD)	Mean (SD)	
Age (years)	39.2 (10.8)	39.6 (11.3)	38.8 (10.3)	
Weight (kg)	71.1 (15.7)	80.6 (13.8)	62.8 (12.0)	<0.001
Height (m)	167.1 (9.4)	173.8 (7.3)	161.3 (6.8)	<0.001
BMI (kg/m^2^)	25.3 (4.6)	26.7 (4.3)	24.1 (4.6)	<0.001
*BMI categories (%)*				
Underweight (BMI < 18.5 kg/m^2^)	9.5	3.1	15.0	<0.001
Normal weight (BMI 18.5-<25 kg/m^2^)	43.7	34.7	51.3	<0.001
Overweight (BMI 25-<30 kg/m^2^)	32.3	43.6	22.8	<0.001
Obese (BMI ≥30 kg/m^2^)	14.5	18.7	10.9	<0.001
BAI (kg/m^2^)	28.7 (5.1)	26.6 (3.9)	30.4 (5.3)	<0.001
Hip circumference	100.4 (9.0)	102.1 (8.0)	98.9 (9.6)	<0.001
Waist circumference	86.6 (13.2)	93.7 (11.8)	80.6 (11.1)	<0.001
% Fat BIO	27.9 (8.2)	23.7 (7.2)	31.6 (7.1)	<0.001

## Results

Age and anthropometric characteristics of participants in the study categorized by gender are shown in Table 1. Significant differences (*p*<0.001) between men and women were found in all anthropometric parameters but in the age of men and women participating in the study. As expected, men were taller, heavier and presented higher BMI values. Taking into account BMI categories, the percentage of subjects in underweight and normal weight categories was significantly higher in women than in men. However, the percentage of obese and overweight subjects was significantly higher in men than in women. BAI values were significantly higher and hip and waist circumferences lower in women than in men. Regarding BIA measurements, % fat determined by BIA was higher in women than in men.

Coefficients of bivariate correlations among BAI, BMI, height, weight, hip and waist circumferences and % fat determined by BIA were calculated. When all the participants were considered together, significant correlations were found for all parameters. BAI and BMI showed a good correlation (*r* = 0.64, *p*<0.001). A strong correlation was found between BAI and the % fat (*r* = 0.74, *p*<0.001), which is even stronger than the one between BMI and % fat (*r* = 0.54, *p*<0.001). Strong correlations were also found between BAI and hip circumference (*r* = 0.65, *p*<0.001) and, also, between BAI and height (*r* = -0.58, *p*<0.001). Correlation between the BMI and weight was much stronger (*r* = 0.85, p<0.001) than the one between BAI and weight (*r* = 0.22, p<0.001) and, also than correlation between % body fat and weight (*r* = 0.22, p<0.001), being these two last correlations very similar. Furthermore, correlation between the BMI and hip circumference were stronger (*r* = 0.82, p<0.001) than the ones obtained when the BAI (*r* = 0.65, p<0.001) and % body fat (*r* = 0.50, p<0.001) were correlated with hip circumference. Regarding waist circumference, correlation between BMI and waist circumference (*r* = 0.85, p<0.001) was much stronger than the ones between the BAI and waist circumference (*r* = 0.37, p<0.001) and between % Fat and waist circumference (*r* = 0.33, p<0.001).

When participants in the study were categorized by gender, some changes were observed in the aforementioned correlations (Table 2 for men and Table 3 for women). Correlation between BAI and BMI categorized by gender were slightly higher (*r* = 0.78 in men and *r* = 0.86 in women, p<0.001) than the one observed for the whole group of participants, but no differences were observed between genders. Correlation between % fat and BMI showed the same Pearson coefficient in men and in women (*r* = 0.80, p<0.001), being this correlation higher than the one observed when men and women were considered together. However, a different pattern between men and women is observed. It seems that for the same BMI, % fat is higher in women than in men. As a consequence of these differences, when men and women participating in this study were considered together, the correlation between % fat and BMI became lower. However, when the same methodology was applied to % body fat and BAI, correlations in men and in women were quite similar, not only between genders (*r* = 0.68 in men and *r* = 0.71 in women, p<0.001 for both), but also when these values, categorized by gender, were compared to the one obtained considering the whole group of participants (*r* = 0.74). However, it should be highlighted that, when categorized by gender, these correlations between BMI and % fat were higher than the ones obtained between BAI and % fat for both men and women. On the other hand, correlations categorized by gender between the BAI and weight (*r* = 0.49 in men and *r* = 0.63 in women, p<0.001) and also between the BAI and hip circumference (*r* = 0.72 in men and *r* = 0.82 in women, p<0.001) were higher than correlations obtained for the whole group of participants.

**Table 2:** Correlation matrix between BAI, BMI, % Fat from BIA, hip and waist circumferences, height, and weight in men.

	BAI	BMI	% Fat	Hip	WC	Height	Weight
BAI	1						
BMI	0.781***	1					
% Fat1	0.678***	0.801***	1				
Hip circumference	0.715***	0.754***	0.619***	1			
Waist circumference	0.688***	0.876***	0.771***	0.810***	1		
Height	-0.494***	-0.146***	-0.170***	0.251***	0.052*	1	
Weight	0.491***	0.870***	0.672***	0.835***	0.851***	0.359***	1

1 % Fat determined by the bioelectrical impedance analysis (BIA). WC: waist circumference.

The level of significance was ^*^p <0.05, ^**^p <0.01, ^***^p <0.001.

**Table 3:** Correlation matrix between BAI, BMI, % Fat from BIA, hip and waist circumferences, height, and weight in women.

	BAI	BMI	% Fat	Hip	WC	Height	Weight
BAI	1						
BMI	0.863^***^	1					
% Fat^1^	0.713^***^	0.804^***^	1				
Hip circumference	0.819^***^	0.859^***^	0.719^***^	1			
Waist circumference	0.691^***^	0.837^***^	0.721^***^	0.802^***^	1		
Height	-0475^***^	-0.174^***^	-0.136^***^	0.112^***^	0.035 ^ns^	1	
Weight	0.628^***^	0.894^***^	0.717^***^	0.888^***^	0.831^***^	0.264^***^	1

1 % Fat determined by the bioelectrical impedance analysis (BIA). WC: waist circumference.

The level of significance was ^*^p <0.05, ^**^p <0.01, ^***^p <0.001.

**Figure 1a:**
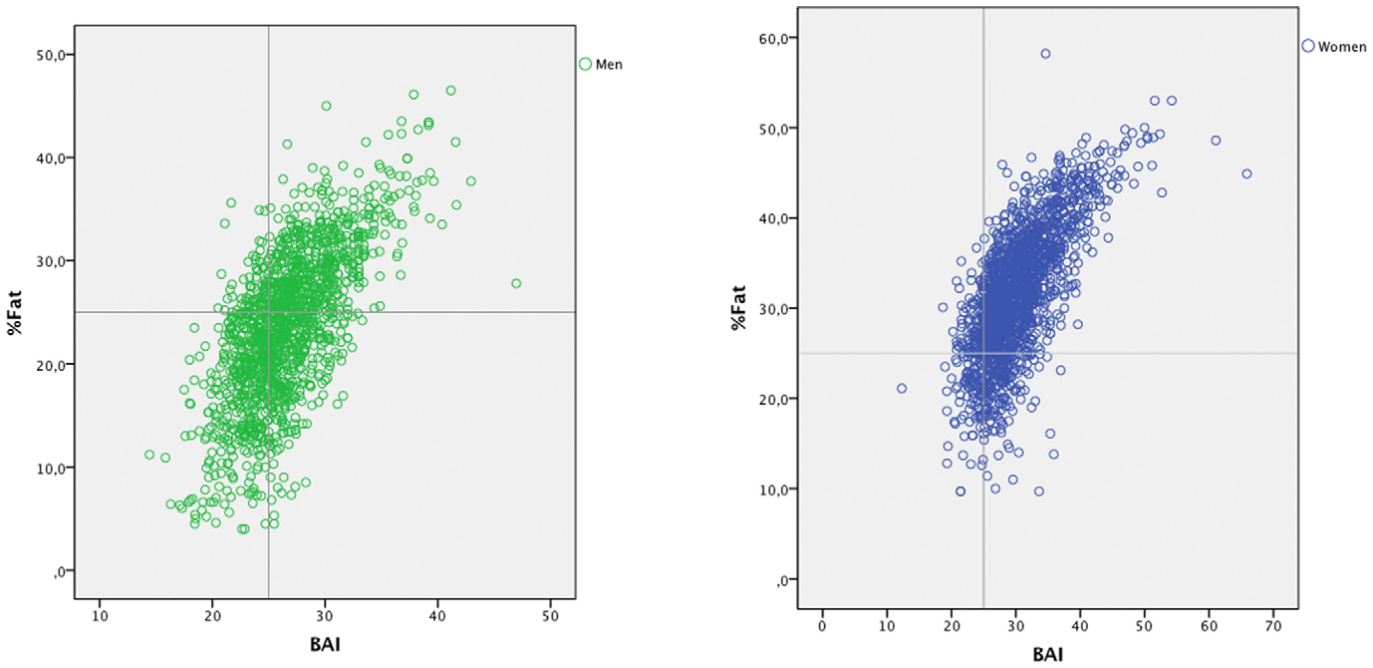
% Body fat (from bioelectrical impedance analysis (BIA)) vs. body adiposity index (BAI) for males. Figure 1b. % Body fat (from bioelectrical impedance analysis (BIA)) vs. body adiposity index (BAI) for females.

The relation between BAI and % fat determined with BIA and the ability to discriminate individuals with higher or lower percentage of fat is shown in Figures 1a (men) and 1b (women). A different behaviour of the BAI in men and women was observed when the ability to discriminate individuals with higher or lower percentage of fat was considered. In this sense, taking the % fat as reference, with the cutoff of 35% for women and 25% for men, it is observed that BAI overestimates obesity in men, using BIA as the reference measurement. On the other hand, in women a slight underestimation could be produced, but in a similar proportion to that of BMI [30]. The cutoff points used in this analysis are the ones suggested for adiposity as a risk factor (25% body fat in males and 35% body fat in females) [31].

**Figure 2a:**
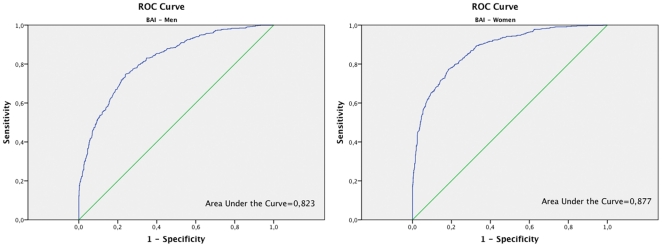
ROC curve analysis for BAI in men. Figure 2b. ROC curve analysis for BAI in women.

To improve the discrimination capacity of BAI, when high values of fat are obtained, respect to the % of fat determined by BIA, the ROC curve was used (Figure 2a for men and 2b for women). In men, the cutoff point value of 27 for the BAI provided a sensitivity of 69% (95% CI: 65–72%), a positive predictive value of 73% (95% CI: 69–76%), specificity of 79% (95%: 77–82%) and negative predictive value of 76% (95% CI: 73–79%). In women the cutoff point of 32 for the BAI provides a sensitivity of 70% (95% CI 66–74%), a positive predicative value of 70% (95% CI 66–74%), specificity of 86% (95%: 84 -88%) and a negative predictive value of 86% (95% CI: 84–88%).

**Figure 3a:**
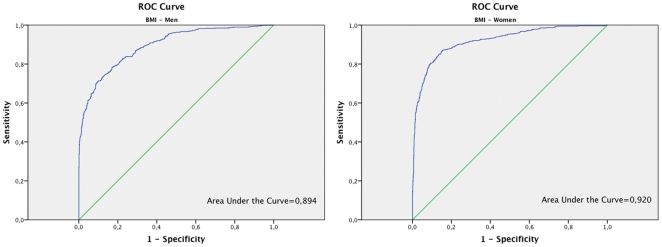
ROC curve analysis for BMI in men. Figure 3b. ROC curve analysis for BMI in women.

ROC curve for BMI was also obtained (Figure 3a for men and 3b for women) and the cutoff point value of 25 was used. Considering this cutoff point, in men sensitivity was 91% (95% CI: 89–94%), positive predictive value of 65%, specificity 61% (95% CI: 58–65%) and negative predictive value 90%. In women, sensitivity was 82% (95% CI: 78–85%), positive predictive value of 81%, specificity 89% (95% CI: 87–91%) and negative predictive value 89%.

## Discussion

To our knowledge this is the first study focused on Caucasian individuals that demonstrates the applicability of BAI as a method to determine adiposity (% body fat) in this population, comparing these values with the ones of BMI and, also, with measures obtained by BIA. Probably, the main finding of the present study is that, in general, the BAI does not overcome the limitations of BMI.

BMI is routinely applied to estimate body fat and to classify overweight and obesity, but has clear well known limitations [32]. The BMI is particularly inaccurate in athletes, who present a high lean body mass [24]. Furthermore, the BMI does not consider the differences between men and women. In addition, taking into account the child growth standards, the BMI is not a good method to classify children according to their fat content, and the most prevalent approach is to use BMI normalized by age, which involves complex mathematical calculations [33]. These, and other, reasons lead to suggest the utilization of a new index, the BAI, which is calculated with the hip circumference and the height (weight is not needed). The BAI measurement requires very simple instrumentation, being very useful in undeveloped or remote places where accurate measurement of weight can be difficult, or scales are not available [7]. This could suppose an important advantage for the BAI over BMI.

Keys et al. reported a high correlation between BMI and adiposity [22]. In the present study, and considering all the participants, the correlation found between BAI and % fat (*r* = 0.74, p<0.001) was higher than the one between the BMI and the % of fat (*r* = 0.54, p<0.001). Given that sex differences in hip circumference and adiposity are large, it has been suggested that hip circumference captures male–female differences in adiposity better than the BMI [7]. Thus, the utilization of hip circumference supposes an important conceptual advantage of the BAI over BMI because differences between men and women regarding adiposity are reflected more properly using the hip circumference than they are considered in the BMI. In fact, correlations between the hip circumference and the % body fat categorized by gender are higher than the one obtained with the whole group of participants. However, when men and women were considered separately, correlation coefficients between BMI and % fat for both men and women were higher than the ones between BAI and % fat. Thus, it can be concluded that one of the limitations of the BAI is that uses the hip circumference as measure of corporal volume or weight, following the same model of perfect cylinder used in BMI, without considering that the human body is not a perfect cylinder [34]. Furthermore, this model does not consider the differences between body-types, as considers all of them based on the same cylinder model. In this sense, the BAI does not improve results obtained using BMI. Correlations taking into account the waist circumference confirmed these observations. Waist circumference is a good indicator of body fat distribution [15]. The higher correlations obtained for both men and women between BMI and the waist circumference than between BAI and the waist circumference could indicate that BMI captures male–female differences in adiposity better than the BMI. Nevertheless, and as with the BMI, waist circumference measurements, in addition to the BAI, will be needed to define the risk associated with between-individual differences in adipose tissue distribution. In fact, BAI shares similar limitations to those that arose for the BMI. More studies should be conducted to establish if the BAI overcomes the well-known limitations of the BMI.

Results obtained regarding correlations are very similar to the ones reported by Bergman in their study focused on the use of BAI in a non-Caucasian population [7]. This observation is very interesting since in the present study the BIA has been used as the reference measure of adiposity and results obtained are similar to the ones obtained using DXA as standard measure [19]. In addition to the technical ones, BIA and DXA show other important differences: BIA is absolutely harmless and is much cheaper than DXA.

A different behaviour of the BAI in men and women when considering the ability to discriminate individuals with higher or lower percentage of fat has been observed in the present study. The sensitivity of BAI to detect an excessive percentage of fat in body composition with respect to BIA is 47% in women and 88% in men. However, the specificity is 86% in women and 60% in men. Therefore, using the BAI there is a significant percentage of men who despite having normal levels of fat would be categorized in the group of excessive fat. Taking into account this observation, a ROC curve was done. This curve was useful in order to allow an improved determination of the best cut-off point showing graphically the tradeoff between sensitivity and specificity. ROC curves represent the rate of true positives versus the rate of false positives and are used to determine the more precise cut-off. In men, the cut-off point of 27 on the BAI provides a sensitivity of 69% and specificity of 79%. In women the cut-off point of 32 provides a sensitivity of 70% and a specificity of 86%. The area under the ROC curve is also considered in the analysis of ROC curves. This area under the ROC curve is a measure of how well a parameter can distinguish between two diagnostic groups. When the ROC curves for BMI and BAI categorized by gender were compared, higher areas under the curve were observed for BMI. Thus, in this sense the discriminatory capacity of the BMI, measured by the area under the ROC curve, is higher than the one of BAI.

In conclusion, results of the present study suggest that the BAI is a good tool to measure adiposity in Caucasian populations both in research and in the clinical practice due, at least in part, to the advantages over other more complex mechanical or electrical systems. Probably, the most important advantage of BAI over BMI is that weight is not needed. However, in general it seems that the BAI does not overcome the limitations of BMI. A different behaviour of the BAI in men and women when considering the ability to discriminate individuals with higher or lower percentage of fat was observed. Changing the cutoff points as indicated previously in men and women, greatly improves the sensitivity and specificity of the BAI. More studies should be performed to investigate if corrections should be included in the BAI measurement as it is when the BMI is used in children. All these determinants could lead to facilitate the introduction of BAI in both clinical practice and research and to introduce it as a predictor of morbidity and mortality.
